# Risk of Congenital Heart Defects after Ambient Heat Exposure Early in Pregnancy

**DOI:** 10.1289/EHP171

**Published:** 2016-08-05

**Authors:** Nathalie Auger, William D. Fraser, Reg Sauve, Marianne Bilodeau-Bertrand, Tom Kosatsky

**Affiliations:** 1 University of Montreal Hospital Research Centre, Montreal, Quebec, Canada; 2 Institut national de santé publique du Québec, Montreal, Quebec, Canada; 3 University of Sherbrooke Hospital Research Centre, Sherbrooke, Quebec, Canada; 4 Departments of Pediatrics and Community Health Sciences, Cummings School of Medicine, University of Calgary, Calgary, Alberta, Canada; 5 National Collaborating Centre for Environmental Health, British Columbia Centre for Disease Control, Vancouver, British Columbia, Canada

## Abstract

**Background:**

Congenital heart defects may be environmentally related, but the association with elevated ambient temperature has received little attention.

**Objectives:**

We studied the relationship between outdoor heat during the first trimester of pregnancy and risk of congenital heart defects.

**Methods:**

We carried out a retrospective cohort study of 704,209 fetuses between 2 and 8 weeks postconception from April to September in Quebec, Canada, 1988–2012. We calculated the prevalence of congenital heart defects at birth according to the number of days women were exposed to maximum temperature ≥ 30°C. In log-binomial regression models, we estimated prevalence ratios (PR) and 95% confidence intervals (CI) for the relationship of temperature with seven critical and eight noncritical heart defects, adjusted for pregnancy characteristics.

**Results:**

Prevalence of congenital heart defects was 979.5 per 100,000 for 10 days or more of temperature ≥ 30°C compared with 878.9 per 100,000 for 0 days of exposure. Temperature was more precisely associated with noncritical than critical defects, which had lower prevalence. Fetuses exposed to 15 days of temperature ≥ 30°C between 2 and 8 weeks postconception had 1.06 times the risk of critical defects (95% CI: 0.67, 1.67) and 1.12 times the risk of noncritical defects (95% CI: 0.98, 1.29) relative to 0 days. Associations were higher for atrial septal defects (PR 1.37, 95% CI: 1.10, 1.70) than for other noncritical defects. For atrial septal defects, associations with elevated temperatures began the 3rd week postconception.

**Conclusions:**

Extreme heat exposure during the first trimester may be associated with noncritical heart defects, especially of the atrial septum.

**Citation:**

Auger N, Fraser WD, Sauve R, Bilodeau-Bertrand M, Kosatsky T. 2017. Risk of congenital heart defects after ambient heat exposure early in pregnancy. Environ Health Perspect 125:8–14; http://dx.doi.org/10.1289/EHP171

## Introduction

Congenital heart defects are a leading congenital anomaly worldwide, occurring in every 8 live births per 1,000 (van der Linde et al. 2011). Most congenital heart defects are noncritical, but critical defects lead to significant morbidity and mortality if not treated promptly after birth (Mahle et al. 2009; Oster et al. 2013). The etiology of heart defects is poorly understood (van der Bom et al. 2011), despite the growing proportion of affected infants who survive to adulthood, and increasing healthcare costs (Ávila et al. 2014). Limited evidence suggests that environmental exposures are risk factors for congenital heart defects (van der Bom et al. 2011), with air pollution linked to coarctation of the aorta, tetralogy of Fallot, and atrial septal defects (Schembari et al. 2014; Vrijheid et al. 2011; Chen et al. 2014). The possibility that elevated temperatures or heat waves increase risk of congenital heart defects has received little attention.

In animal studies, elevated temperature is a proven cardiac teratogen (Edwards 2006). In humans, high temperature from fever in the first trimester is associated with congenital heart defects in offspring (Dreier et al. 2014; Shi et al. 2014; Tikkanen and Heinonen 1991). Despite this evidence, only two studies have considered the possibility that ambient heat exposure is related to congenital heart defects. One found no association in a case–control analysis of 6,422 infants with birth defects of all kinds (Van Zutphen et al. 2012), and the other a weak association with atrial but not ventricular septal defects in a cohort study of 135,527 neonates (Agay-Shay et al. 2013). The studies were either underpowered or did not evaluate heart defects comprehensively (Agay-Shay et al. 2013; Van Zutphen et al. 2012), leaving it unclear whether ambient heat exposure affects risk.

Climate change is projected to increase mean global temperatures during this century, including the frequency and intensity of heat waves (McCoy and Hoskins 2014). Our objective was to determine the relationship between elevated temperatures during pregnancy and risk of congenital heart defects in offspring.

## Methods

We extracted data from the Maintenance and Use of Data for the Study of Hospital Clientele database (Ministère de la Santé et des Services sociaux 2016) a compilation of hospital discharge abstracts containing all deliveries in Quebec, a large Canadian province (Auger et al. 2015). We linked discharge abstracts for all women who delivered in hospital with abstracts for the neonate. We used a retrospective cohort design, analyzing fetuses between 2 and 8 weeks postconception from the months of April to September, 1988–2012. We selected these months because in Quebec heat waves or elevated temperatures do not occur between October and March.

### Inclusion Criteria and Exposure Window

We used the period spanning 2–8 weeks postconception as the exposure window, based on evidence that cardiogenesis is completed during this time (Moorman et al. 2003). We first derived the conception date using the date and gestational age recorded at delivery (Schembari et al. 2014). We identified all pregnancies conceived between the middle of March and beginning of August, ensuring that the exposure window fell within the months of April through September. To be included, women had to be at least 2 weeks postconception the first week of April or have reached 8 weeks postconception by the last week of September. Thus, pregnancies at 3 weeks or more at the start of April were not included, since week 2 would have been in March or February. Similarly, women between 2 and 7 weeks postconception at the end of September were not included. We did so to ensure that all women had complete exposure data.

We excluded infants with chromosomal anomalies, as genetic disorders occur at conception, not during fetal development. We could not include pregnancies that miscarried, were terminated, or resulted in stillbirth, as we did not have information on congenital defects for these fetuses.

### Temperature Exposure

We obtained temperature data from Environment Canada (Environment Canada 2015) for the 18 meteorological stations representative of each health region in Quebec. The main exposure was defined as the number of days with maximum temperature ≥ 30°C during the exposure window, analyzed continuously and categorically (0, 1–2, 3–9, ≥ 10 days). Although 30°C is an arbitrary cutoff, a higher threshold of 32°C resulted in few fetuses exposed, and a lower cutoff diluted the associations. A threshold of 30°C provided a meaningful balance of high temperatures with enough days and pregnancies to show effects. Because the exposure window extends over 6 weeks, we did not use average measures of temperature which mask variation in exposure. We nonetheless assessed the maximum temperature of each week during the exposure window as a separate continuous exposure, without imposing a temperature threshold. We obtained data on percent relative humidity for all meteorological stations.

### Cardiac Defects

We defined heart defects as structural anomalies anatomically located in the cardiac region, not including conduction defects or cardiomyopathy (Hoffman and Kaplan 2002). We included seven critical defects (tetralogy of Fallot; transposition of great vessels, including double outlet right and left ventricle; truncus arteriosus; hypoplastic left heart; common ventricle; coarctation of aorta, including interrupted aortic arch; other critical defects including total anomalous pulmonary venous return, Ebstein’s anomaly, and tricuspid and pulmonary atresia) (Oster et al. 2013), and eight noncritical defects (endocardial cushion; ventricular septum; atrial septum; valves; aorta; pulmonary artery; heterotaxy; other). In addition, we evaluated heart defects by site (aorta or pulmonary artery; valves; septum) and presence of single vs. multiple defects. We identified heart defects using diagnostic codes of the *International Classification of Diseases* (ICD) (Canadian Institute for Health Information 2009; WHO 1979), and procedure codes for repair of select defects (Auger et al. 2015). The ICD is the most commonly used system to classify heart defects, with 88% sensitivity to detect cases in Quebec (Beauséjour Ladouceur et al. 2016). In this study, defects were diagnosed during ultrasound examinations *in utero*, or postnatally before discharge from hospital. Heart defects were documented on the infant’s hospital discharge abstract.

### Covariates

We measured possible confounders, including maternal age (< 25, 25–34, ≥ 35 years), comorbidity (preexisting diabetes, hypertension, or heart disease; obesity; blood, thyroid, epilepsy or mood disorders; connective tissue diseases; tobacco or substance abuse) (Auger et al. 2015), parity (0, 1, ≥ 2, unknown), multiple birth, socioeconomic area deprivation (Auger et al. 2015), and month and year of conception in cubic splines. These data were obtained from maternal discharge abstracts.

### Data Analysis

We computed prevalence rates and estimated the association between the number of days with maximum temperature ≥ 30°C and congenital heart defects. We used log-binomial regression models to compute prevalence ratios and 95% confidence intervals (CI) for the association between heart defects and temperature, adjusted for humidity, age, comorbidity, parity, multiple birth, socioeconomic deprivation, month, and period. We used generalized estimating equations with robust error estimators to account for clustering in women with more than one infant (Liang and Zeger 1986), and a Poisson distribution to facilitate convergence of regression models (Zou 2004). We modeled the number of days with temperature ≥ 30°C as a continuous exposure relative to 0 days, using cubic splines with knots at the 5th, 50th, and 95th percentiles (Durrleman and Simon 1989). Similarly, we used splines to model maximum weekly temperature as a continuous exposure relative to 20°C, a comfortable outdoor temperature used in previous research (Auger et al. 2014).

We carried out several sensitivity analyses. We began by replicating analyses after including fetuses whose exposure windows were only partly between April and September. For example, we included fetuses that had not yet reached 2 weeks postconception at the start of April, but were between 3 and 8 weeks. Next, we restricted the exposure window to the months of June, July, and August when temperatures were warmest. We further analyzed isolated atrial septal defects separately, in the event that some were mild cases that were overdetected and would have resolved during infancy (Geva et al. 2014). Finally, we evaluated singleton apart from multiple pregnancies, and excluded women with comorbidity.

We used SAS PROC GENMOD and PROC IML (version 9.3; SAS Institute Inc.) for statistical analysis. The data were anonymous, and we obtained an ethics waiver from the institutional review board of the University of Montreal Hospital Centre.

## Results

There were 704,209 fetuses with exposure windows between April and September, including 6,482 with heart defects at birth (Table 1). Prevalence of critical heart defects was 76.5 per 100,000 (95% CI: 70.2, 83.3) and noncritical heart defects 843.9 per 100,000 (95% CI: 822.7, 865.6). The prevalence of critical and noncritical defects increased with greater number of days of temperature ≥ 30°C during the exposure window. With 10 or more days of exposure, there were 94.8 critical (95% CI: 67.4, 129.6) and 916.3 (95% CI: 826.5, 1013.2) noncritical heart defects per 100,000.

**Table 1:** Pregnancy characteristics and prevalence of critical and noncritical heart defects, 2–8 weeks postconception, Quebec, April through September, 1988–2012.

Characteristic	Total no. of infants	Critical defect		Noncritical defect	
		No. of infants with defects	Prevalence per 100,000 (95% confidence interval)	No. of infants with defects	Prevalence per 100,000 (95% confidence interval)
No. of days ≥ 30°C^*a*^
0 days	218,337	172	78.8 (67.4, 91.5)	1,753	802.9 (765.9, 841.2)
1–2 days	182,430	141	77.3 (65.1, 91.1)	1,552	850.7 (809.1, 893.9)
3–9 days	262,300	187	71.3 (61.4, 82.3)	2,261	862.0 (827.0, 898.1)
≥ 10 days	41,142	39	94.8 (67.4, 129.6)	377	916.3 (826.5, 1013.2)
Age, years
< 25	150,747	124	82.3 (68.4, 98.1)	1,247	827.2 (782.1, 874.2)
25–34	460,957	333	72.2 (64.7, 80.4)	3,777	819.4 (793.6, 845.8)
≥ 35	92,505	82	88.6 (70.5, 110.0)	919	993.5 (930.5, 1059.5)
Parity
0	391,891	295	75.3 (66.9, 84.4)	3,364	858.4 (829.8, 887.8)
1	224,711	168	74.8 (63.9, 87.0)	1,801	801.5 (765.0, 839.2)
≥ 2	80,343	69	85.9 (66.8, 108.7)	702	873.8 (810.6, 940.5)
Comorbidity^*b*^
No	640,346	436	68.1 (61.8, 74.8)	4,840	755.8 (734.8, 777.4)
Yes	63,863	103	161.3 (131.7, 195.6)	1,103	1727.1 (1627.5, 1831.2)
Multiple birth
No	685,147	509	74.3 (68.0, 81.0)	5,430	792.5 (771.7, 813.8)
Yes	19,062	30	157.4 (106.2, 224.6)	513	2691.2 (2466.1, 2930.9)
Socioeconomic deprivation^*c*^
No	525,114	396	75.4 (68.2, 83.2)	4,328	824.2 (799.9, 849.0)
Yes	139,588	115	82.4 (68.0, 98.9)	1,320	945.6 (895.5, 997.8)
Period
1988–1995	244,545	146	59.7 (50.4, 70.2)	1,590	650.2 (618.7, 682.8)
1996–2003	213,761	159	74.4 (63.3, 86.9)	2,164	1012.3 (970.3, 1055.7)
2004–2012	245,903	234	95.2 (83.4, 108.2)	2,189	890.2 (853.4, 928.1)
Total	704,209	539	76.5 (70.2, 83.3)	5,943	843.9 (822.7, 865.6)
^***a***^Number of days with maximum daily temperature ≥ 30°C during weeks 2–8 postconception. ^***b***^Preexisting diabetes, hypertension, or heart disease; obesity; blood, thyroid, epilepsy or mood disorders; connective tissue diseases; tobacco or substance abuse. ^***c***^Poorest fifth of the population for socioeconomic area data on median personal income, proportion of the population with no high school diploma, and unemployment rate.					

Compared with 0 days of temperature ≥ 30°C, fetuses with ≥ 10 days of exposure had higher prevalence of three critical defects, including transposition of great vessels (29.2 vs. 19.2 per 100,000), truncus arteriosus (12.2 vs. 5.5 per 100,000) and coarctation of aorta (21.9 vs. 16.5 per 100,000), but prevalence was not elevated for other critical defects (Table 2). Among noncritical defects, fetuses with ≥ 10 days of exposure had more defects of the atrial septum (413.2 vs. 289.0 per 100,000), aorta (19.4 vs. 11.9 per 100,000), heterotaxy (14.6 vs. 8.2 per 100,000) and other defects (255.2 vs. 223.0 per 100,000). Both single and multiple defects were more common in fetuses exposed to 10 days or more of temperature ≥ 30°C, compared with 0 days of exposure. The trend over increasing temperature was most apparent for noncritical defects, especially atrial septal defects.

**Table 2:** Prevalence of congenital heart defects according to ambient temperature 2–8 weeks postconception, Quebec, April through September, 1988–2012.

Type of heart defect	No. of days ≥ 30°C^*a*^								
	0 days (*N *= 218,337)		1–2 days (*N *= 182,430)		3–9 days (*N *= 262,300)		≥ 10 days (*N *= 41,142)		Trend *p*-value^*b*^
	No. infants with defects	Prevalence per 100,000 (95% CI)	No. infants with defects	Prevalence per 100,000 (95% CI)	No. infants with defects	Prevalence per 100,000 (95% CI)	No. infants with defects	Prevalence per 100,000 (95% CI)	
Critical defect
Tetralogy of Fallot	44	20.2 (14.6, 27.1)	32	17.5 (12.0, 24.8)	53	20.2 (15.1, 26.4)	9	21.9 (10.0, 41.5)	0.8
Transposition of great vessels	42	19.2 (13.9, 26.0)	33	18.1 (12.5, 25.4)	37	14.1 (9.9, 19.4)	12	29.2 (15.1, 50.9)	0.8
Truncus arteriosus	12	5.5 (2.8, 9.6)	5	2.7 (0.9, 6.4)	14	5.3 (2.9, 9.0)	5	12.2 (3.9, 28.4)	0.3
Hypoplastic left heart	24	11.0 (7.0, 16.4)	18	9.9 (5.8, 15.6)	31	11.8 (8.0, 16.8)	< 5	4.9 (0.6, 17.6)	0.8
Common ventricle	6	2.7 (1.0, 6.0)	8	4.4 (1.9, 8.6)	6	2.3 (0.8, 5.0)	< 5	2.4 (0.1, 13.5)	0.7
Coarctation of aorta	36	16.5 (11.5, 22.8)	38	20.8 (14.7, 28.6)	39	14.9 (10.6, 20.3)	9	21.9 (10.0, 41.5)	0.9
Other	19	8.7 (5.2, 13.6)	12	6.6 (3.4, 11.5)	19	7.2 (4.4, 11.3)	< 5	7.3 (1.5, 21.3)	0.6
Any	172	78.8 (67.4, 91.5)	141	77.3 (65.1, 91.1)	187	71.3 (61.4, 82.3)	39	94.8 (67.4, 129.6)	0.9
Noncritical defect
Endocardial cushion	33	15.1 (10.4, 21.2)	34	18.6 (12.9, 26.0)	40	15.2 (10.9, 20.8)	5	12.2 (3.9, 28.4)	0.8
Ventricular septum	617	282.6 (260.8, 305.8)	546	299.3 (274.7, 325.4)	755	287.8 (267.7, 309.1)	116	282.0 (233.0, 338.1)	0.9
Atrial septum	631	289.0 (266.9, 312.4)	584	320.1 (294.7, 347.1)	867	330.5 (308.9, 353.3)	170	413.2 (353.5, 480.0)	0.0001
Valve	62	28.4 (21.8, 36.4)	73	40.0 (31.4, 50.3)	99	37.7 (30.7, 45.9)	13	31.6 (16.8, 54.0)	0.2
Aorta	26	11.9 (7.8, 17.4)	22	12.1 (7.6, 18.3)	43	16.4 (11.9, 22.1)	8	19.4 (8.4, 38.3)	0.1
Pulmonary artery	174	79.7 (68.3, 92.4)	163	89.3 (76.2, 104.2)	230	87.7 (76.7, 99.8)	29	70.5 (47.2, 101.2)	0.8
Heterotaxy	18	8.2 (4.9, 13.0)	17	9.3 (5.4, 14.9)	20	7.6 (4.7, 11.8)	6	14.6 (5.4, 31.7)	0.7
Other	487	223.0 (203.7, 243.7)	384	210.5 (190.0, 232.6)	620	236.4 (218.1, 255.7)	105	255.2 (208.8, 308.9)	0.1
Any	1,753	802.9 (765.9, 841.2)	1,552	850.7 (809.1, 893.9)	2,261	862.0 (827.0, 898.1)	377	916.3 (826.5, 1013.2)	0.007
No. of defects
Single	1,613	738.8 (703.3, 775.6)	1,399	766.9 (727.3, 808.0)	2,050	781.5 (748.2, 816.0)	323	785.1 (702.1, 875.1)	0.09
Multiple	306	140.2 (124.9, 156.8)	282	154.6 (137.1, 173.7)	391	149.1 (134.7, 164.6)	80	194.4 (154.2, 242.0)	0.07
Any	1,919	878.9 (840.2, 919.0)	1,681	921.4 (878.1, 966.4)	2,441	930.6 (894.2, 968.1)	403	979.5 (886.6, 1079.5)	0.02
^***a***^Number of days with maximum daily temperature ≥ 30°C during weeks 2–8 postconception. ^***b***^Cochran–Armitage trend test.									

**Figure 1:**
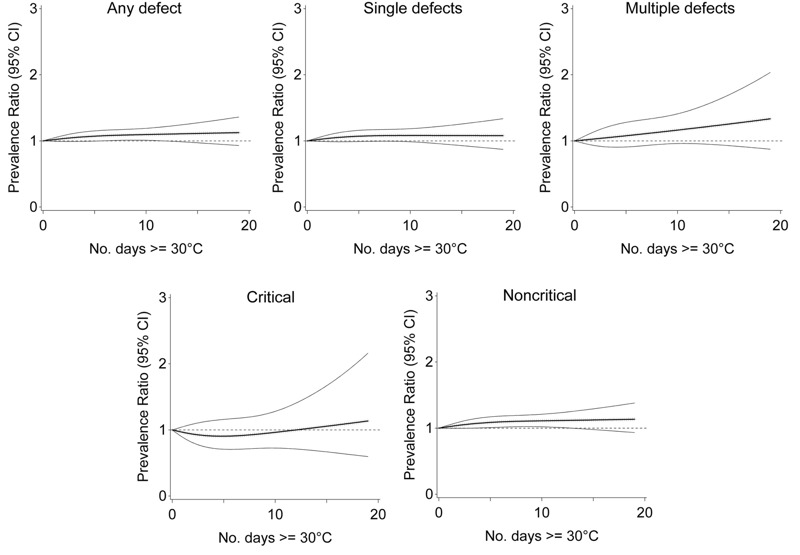
Association between ambient temperature 2–8 weeks postconception and congenital heart defects, Quebec, April through September, 1988–2012. Note: Prevalence ratio (bold central line) and 95% CI (light outer bands) for number of days with maximum temperature ≥ 30°C between weeks 2 and 8 postconception, relative to 0 days, adjusted for maternal age, comorbidity, parity, multiple birth, socioeconomic area deprivation, month and period of conception, and humidity.

In regression models adjusted for relative humidity and pregnancy characteristics, exposure to progressively more days with maximum temperature ≥ 30°C was associated with noncritical but not critical heart defects (Figure 1). Relative to 0 days, exposure to 15 days of temperature ≥ 30°C was associated with 1.12 times the risk of noncritical defects (95% CI: 0.98, 1.29), 1.06 times the risk of critical defects (95% CI: 0.67, 1.67), 1.08 times the risk of single defects (95% CI: 0.93, 1.26), and 1.26 times the risk of multiple defects (95% CI: 0.93, 1.70). For noncritical defects, the associations increased progressively with greater number of days exposed.

**Figure 2:**
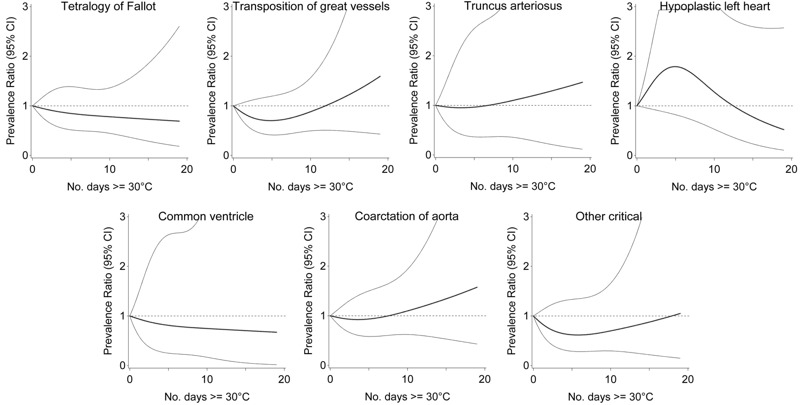
Association between ambient temperature 2–8 weeks postconception and critical heart defects, Quebec, April through September, 1988–2012. Note: Prevalence ratio (bold central line) and 95% CI (light outer bands) for number of days with maximum temperature ≥ 30°C between weeks 2 and 8 postconception, relative to 0 days, adjusted for maternal age, comorbidity, parity, multiple birth, socioeconomic area deprivation, month and period of conception, and humidity.

**Figure 3:**
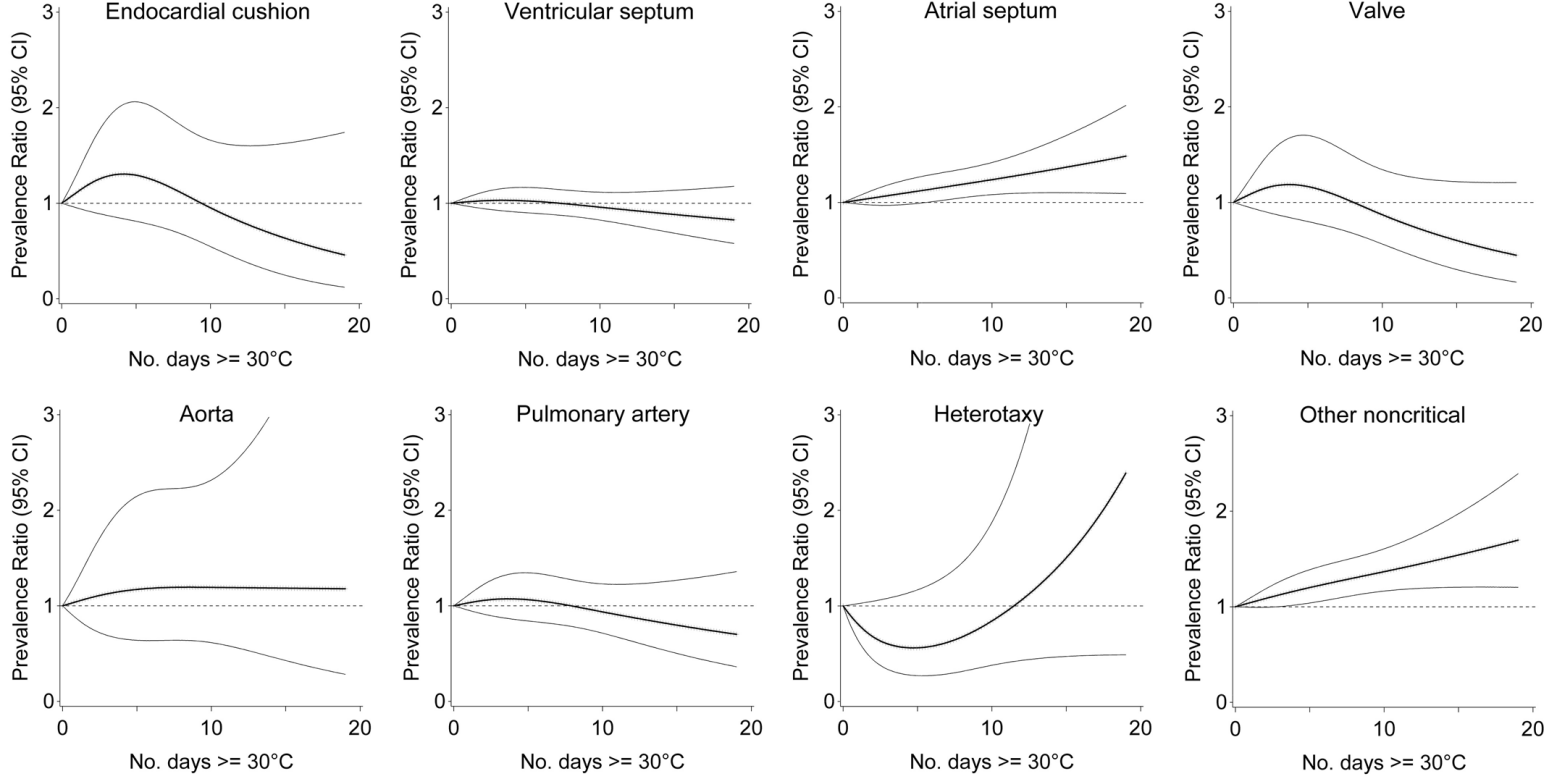
Association between ambient temperature 2–8 weeks postconception and noncritical heart defects, Quebec, April through September, 1988–2012. Note: Prevalence ratio (bold central line) and 95% CI (light outer bands) for number of days with maximum temperature ≥ 30°C between weeks 2 and 8 postconception, relative to 0 days, adjusted for maternal age, comorbidity, parity, multiple birth, socioeconomic area deprivation, month and period of conception, and humidity.

The number of days with temperature ≥ 30°C was not significantly associated with any specific critical heart defect in adjusted models, however prevalence was low and positive associations tended to be present with transposition of the great vessels, truncus arteriosus and coarctation of the aorta (Figure 2). However, the number of days with temperature ≥ 30°C was associated with select noncritical defects, including atrial septal and other noncritical defects (Figure 3). Relative to 0 days, exposure to 15 days of temperature ≥ 30°C was associated with 1.37 times the risk of atrial septal defects (95% CI: 1.10, 1.70), and 1.54 times the risk of other noncritical defects (95% CI: 1.20, 1.97). The associations increased steadily with more days exposed.

**Figure 4:**
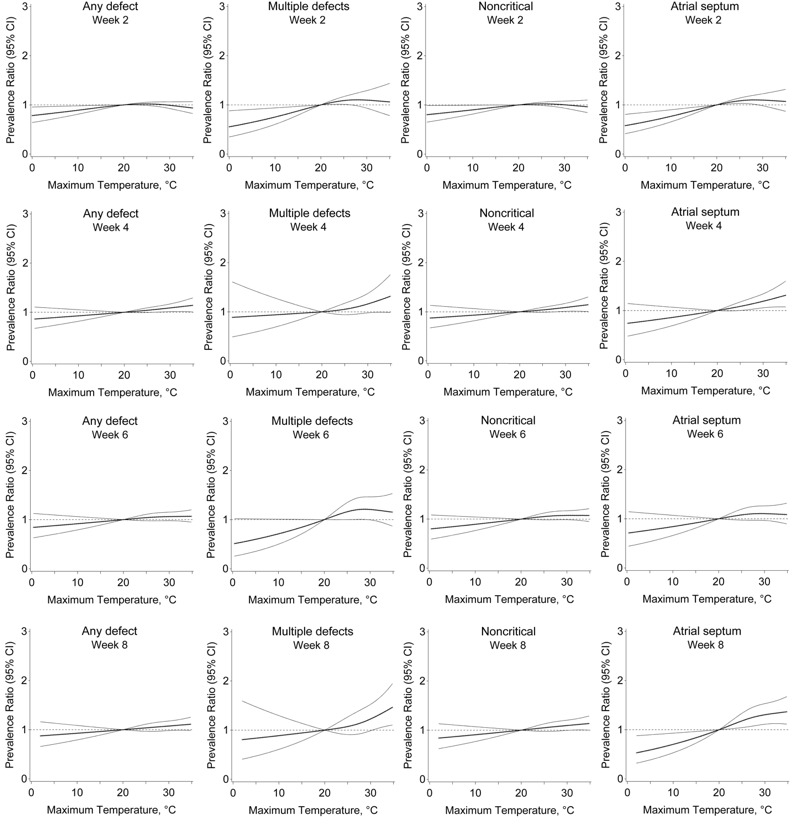
Association between maximum weekly temperature 2–8 weeks postconception and congenital heart defects, Quebec, April through September, 1988–2012. Note: Prevalence ratio (bold central line) and 95% confidence interval (light outer bands) for maximum temperature during each week postconception, relative to 20°C, adjusted for maternal age, comorbidity, parity, multiple birth, socioeconomic area deprivation, month and period of conception, and humidity. Associations at weeks 3, 5, and 7 are not shown to conserve space, but reflect the same trend.

When each week of the exposure window was examined, maximum weekly temperature was associated with single, multiple, and noncritical defects, including atrial septal defects (Figure 4). For single defects, prevalence ratios were strongest week 7, with 32°C associated with 1.13 times (95% CI: 1.01, 1.26) the risk relative to 20°C. Maximum temperatures of 32°C were significantly associated with multiple defects week 8, with 1.31 times (95% CI: 1.04, 1.65) the risk compared with 20°C. For any noncritical defect, maximum weekly temperatures of 32°C were associated with increased risk beginning week 4, with 1.15 times (95% CI: 1.04, 1.27) the risk relative to 20°C the 7th week. Associations with atrial septal defects began to be apparent the 3rd week postconception. Relative to 20°C, temperatures of 32°C were associated with 1.21 times (95% CI: 1.05, 1.40) the risk of atrial septal defects week 3, and 1.33 times (95% CI: 1.13, 1.57) the risk week 8.

In sensitivity analyses, regression models in which we included an additional 224,561 fetuses with exposure windows only partly in April or September yielded slightly attenuated results. When we restricted the exposure window to June through August, the hottest period of the year, the associations strengthened but confidence intervals were wider. For example, exposure to 15 days of temperature ≥ 30°C was associated with 1.76 times the prevalence of multiple defects (95% CI: 1.09, 2.82) relative to 0 days of exposure. Comparison of singleton and multiple pregnancies did not produce conclusive differences, nor did exclusion of women with comorbidity. When we analyzed isolated and nonisolated atrial septal defects separately, there was no clear difference between groups. Finally, alternate knot locations in splines yielded similar results.

## Discussion

Using population-based data for a significant number of fetuses in the first trimester during summer months in a large Canadian province, we demonstrate that greater exposure to maximum daily temperatures ≥ 30° was associated with increased risk of multiple and noncritical congenital heart defects, particularly atrial septal defects. The associations strengthened progressively with more days of exposure to temperatures ≥ 30°C. Associations with elevated temperatures began the 3rd week postconception for atrial septal defects, and were strongest for multiple and noncritical defects towards the 8th week postconception. There was little evidence of an association with critical heart defects, though prevalence was low. This study therefore provides preliminary evidence of a possible influence of extreme summertime heat on prevalence of noncritical heart defects. These results are timely in light of evidence that temperature and heat waves will increase in intensity during this century (McCoy and Hoskins 2014), and highlight a possible impact of heat on fetal heart development.

Few studies have evaluated the association between high temperatures and heart defects (Agay-Shay et al. 2013; Van Zutphen et al. 2012). In New York, a case-control analysis of 6,422 infants with anomalies found no increase in risk of congenital heart defects for fetuses exposed to heat between the 3rd and 8th weeks postconception for at least 1 week in June, July, or August (Van Zutphen et al. 2012). The study however included infants who were only partly exposed during those months, and may have been underpowered. When we included infants who were only partly exposed during summer, the results in our own analysis were attenuated. In Israel, a cohort study of 135,527 infants found an association between temperature and multiple heart defects, including a weak association with isolated atrial septal but not ventricular septal defects (Agay-Shay et al. 2013). Other defects were not examined. Israel has a Mediterranean climate with warm summers and mild winters, and therefore a population resilient to heat. In our population of women less acclimatized to high temperatures, we found a stronger association for atrial septal defects than for multiple defects. The similarity between results in the two studies is nonetheless compelling.

Some studies have considered other sources of heat in relation to heart defects. Maternal fever during first trimester has received the most attention (Dreier et al. 2014), with studies suggesting associations with atrial septal defects and hypoplastic left heart (Tikkanen and Heinonen 1991), and others with ventricular septal and right obstructive defects (Shi et al. 2014). Studies of hot tub or sauna use have reported no association with heart defects (Judge et al. 2004; Tikkanen and Heinonen 1991). Finally, other researchers have proposed that temperature elevations from radiation emitted by ultrasound imaging may cause damage when intensity is high, but an association with congenital heart defects has not been shown (Ziskin and Morrissey 2011).

Mechanisms potentially linking temperature with heart defects are unclear, but include a direct effect of heat, or possible mediators implicated in a heat stress response. Some studies report that exposures to high temperature may cause cell death in fetuses, subsequently resulting in congenital anomalies (Bennett 2010). Cellular death may also occur if elevated temperatures cause placental insufficiency or trigger a heat-shock response. The heat-shock response is a protective cellular mechanism which blocks transcription and translation of normal proteins and increases translation of heat-shock proteins, and may be triggered through multiple routes. While the heat-shock response typically is short lived, interruption of normal biochemical and molecular events during crucial periods of development could have implications for the fetus. In rat models, the duration of the heat-shock response is correlated with risk of heart defects due to heat (Bennett 2010). Elevated ambient temperatures are unlikely to cause a severe heat-shock response in pregnant women, but heat waves have the potential to trigger mild responses with a plausible impact on noncritical or atrial septal defects, especially beginning the 3rd week postconception.

Air pollution is another potential contributor to the associations. Several studies suggest air pollution is associated with congenital heart defects, though most do not account for temperature (Schembari et al. 2014; Vrijheid et al. 2011; Chen et al. 2014). The causal pathways linking temperature and air pollution are however complex and have yet to be fully elucidated (Buckley et al. 2014). Air pollution has potential to mediate or possibly confound associations with temperature. Although pollutants such as ozone that are downstream in the causal pathway may not require adjustment (Buckley et al. 2014), this may not be the case for other pollutants. We did not have information on air pollution, but some data suggest that pollutants have little impact on the relationship between temperature and risk of early delivery in Montreal (Auger et al. 2014). Future research would benefit from disentangling the role of air pollution in the relationship between temperature and congenital heart defects. Similarly, there may be a relationship between temperature and seasonality in congenital heart defects (Caton 2012), also worth exploring in future research.

A separate issue is that mild heart defects, especially of the atrial septum, can be overdetected, particularly for infants born preterm who are under increased surveillance and whose hearts have yet to fully mature (Tanner et al. 2005). Moreover, mild defects can sometimes resolve during infancy or childhood (Geva et al. 2014). However, evidence from existing research suggests that minor defects are not fully explained by overdetection (Tanner et al. 2005), and that high temperature is not associated with preterm birth in Quebec (Auger et al. 2014). Together, these findings make it unlikely that overdetection accounts for the results of our study. Moreover, any overdetection would be present for all fetuses regardless of temperature, resulting in associations that are attenuated but still valid.

This study had limitations. We were restricted to the 9th and 10th revisions of the ICD to classify congenital heart defects. We could not identify congenital heart defects that were diagnosed later in infancy or childhood, though we have no reason to suspect that any missed cases would vary with temperature. Thus, misclassification is likely nondifferential and the association between temperature and congenital heart defects is potentially attenuated. We were limited by the lack of data on indoor temperatures or possible urban heat islands (Agay-Shay et al. 2013). The impact of outdoor heat may be mitigated by air conditioning or worsened in very urban areas that retain heat. Due to limited sample size, we could not evaluate heat waves, often defined in Canada as three consecutive days of temperatures reaching 32°C (Smoyer-Tomic et al. 2003). Critical heart defects were rare, lowering statistical power for this outcome. We did not have data on miscarriages, terminations, or stillbirths, a proportion of which may be due to congenital heart defects. However, recent evidence suggests no association between temperature and risk of stillbirth due to congenital anomalies in Quebec (Auger et al. 2016). We adjusted for maternal co-morbidity, but cannot rule out residual confounding. We did not have information on ethnicity, though Quebec is a diverse multicultural population. Generalizability to other populations remains to be evaluated.

## Conclusions

In this study, we found a positive relationship between the number of days fetuses were exposed to maximum daily temperatures ≥ 30°C and the risk of congenital heart defects. A greater number of hot days was associated with noncritical defects, particularly of the atrial septum, but not critical defects. Associations with maximum weekly temperature began the 3rd week postconception for atrial septal defects. Further study is merited to verify these findings in other populations. Climate change is predicted to increase the frequency and intensity of heat waves, and the impact on risk of congenital heart defects may not be benign.
